# Supplementary material for: Excess mortality during the COVID-19 pandemic (2020–2021) in an urban community of Bangladesh
Source: PLOS Glob Public Health. 2023 Jul 14;3(7):e0002176. doi: 10.1371/journal.pgph.0002176 (PMC10348530; doi:10.1371/journal.pgph.0002176)
Supplement: S1 Table — (DOCX) [file pgph.0002176.s001.docx]

**S1 Table.** The difference in the monthly average number of deaths between pre-COVID-19 and COVID periods, as well as a model-based estimate of the impact of COVID-19.

| **Number of deaths** | **Pre-COVID**  **(Jan 2015 to Feb 2020)** | **During COVID**  **(Mar 2020 to Dec 2021)** | **Difference^1^** | **Relative effect**  **95% Credible Interval^2^** |
| --- | --- | --- | --- | --- |
| Total | 80.8 | 104.2 | 23.4* | 15% (-8.8%, 40%) |
| Male | 48.3 | 64.1 | 15.8* | 27% (-16%, 75%) |
| Female | 32.4 | 40.0 | 7.6* | 6.6% (-50%, 67%) |

^1^Difference between the monthly average deaths, ^2^BSTS model based results on the impact of COVID-19

*p<0.001, based on Wilcoxon rank-sum test
