# Supplementary material for: Excess mortality during the COVID-19 pandemic (2020–2021) in an urban community of Bangladesh
Source: PLOS Glob Public Health. 2023 Jul 14;3(7):e0002176. doi: 10.1371/journal.pgph.0002176 (PMC10348530; doi:10.1371/journal.pgph.0002176)
Supplement: Attachment — Submitted filename: Response to Reviewer_15 April 2023.docx [file pgph.0002176.s002.docx]

**Reviewer #1:**

This is an interesting and important article examining excess mortality during the COVID-19 pandemic in Bangladesh. Authors use both traditional comparisons as well as a Bayesian time series model to examine the all cause mortality in a single cemetery in Bangladesh. The study reports no significant change in morality during the pandemic and describes results in the context of the setting and data sources. A few points should be clarified to provide additional methodological information and highlight study limitations.

**Response:** We appreciate you taking the time and making the effort to evaluate this manuscript and provide insightful feedback. These have significantly contributed to the improvement of the manuscript's overall quality and readability.

Major:
1. Lines 96-97, given this study is only examining cemetery burials in a specific town the assumption that individuals are buried in their local community—and that this practice did not change during the pandemic— has important implications for the results. I would request that the authors acknowledge this limitation more strongly in their discussion. I also request that authors highlight more strongly that these data have not been validated for use of excess mortality estimates in this setting.

**Response:** We have revised the manuscript accordingly (Line 307-309):

“It should be noted that this dataset has not been validated for estimating excess mortality in this specific setting. Therefore, it is essential to exercise caution when interpreting the results and to consider the limitations and potential biases of the data.”

2. The authors use a BSTS interventional time series model, which seems appropriate for the aim. However, additional details in the methods regarding the model selection—including the functional form used in the Bayesian model, what model predictors were included in the forecasting of the counterfactual, etc. I would ask authors to provide more detailed information on the modelling process and factors that were included in the model.

**Response**: Thank you. In response to your suggestion, we have revised the manuscript to include a description of the model, the functional form, and the building strategy (see Methodology section, Line 133-207 ).

3. While the Wilcoxon rank-sum tests indicate a significant difference between the pre-pandemic and pandemic periods, these are not congruent with the regression analyses. In both the sex-specific and overall regression analyses the credible intervals cross 0 and are extremely wide. While the authors indicate that the regression results are not statistically significant, they assert in the abstract that COVID-19 did contribute to excess mortality. This is well described in the discussion, and I would ask authors to use similar language in their abstract.

**Response**: We appreciate your suggestion. Abstract has been revised.

4. Similarly, in the discussion I would ask authors to unpack this further and provide additional context, given that the model-based estimates indicate that there could have been an increase or decrease (based on the wide confidence intervals). Perhaps adding additional context in the limitation on this dataset, particularly since it has not be validated, would be helpful.

The authors point about road traffic accidents decreasing and this offsetting COVID-19 related deaths, is important, and warrants further attention. Authors may also comment on the role of vaccines.

**Response:** We have added additional context and discussion points in the revised manuscript (see Introduction and Discussion section) as well as updated the limitation section. Road accident related deaths and vaccination issues are discussed (Line 273-287).

Minor:
The manuscript could benefit from an additional read through for grammatical and spelling errors and to provide clearer context, some of these instances are highlighted below.
1. Lines 54-55, please revise this sentence – perhaps stating “increase in all-cause morality during the COVID-19 pandemic” instead of using the word including.

**Response:** This sentence has been revised.

2. Line 68, could authors provide some context on what they mean by “average urban setting”

**Response:** This sentenced has been revised.

3. Line 82 – I believe authors mean ‘census data’ not consensus data.

**Response:** Thank you. We have corrected this unintentional typo in the revised manuscript.

4. This is perhaps a minor point, but the authors switch between outbreak, epidemic, and pandemic throughout the manuscript. Please be consistent in how these are used.

**Response:** We have thoroughly reviewed and corrected the manuscript and chosen to use the term "pandemic" throughout.

Additional methodological minor comments are included below

5. Lines 104-105, it is unclear why authors have excluded those under 35. This seems like it would be an important demographic to see differences, in particular since authors note that official records were not reliable during this period. I would request authors provide further context on why they excluded this, or to conduct a sensitivity analysis by including these individuals.

**Response**: Our analysis has only looked at people who are 35 years old or older because this age group has the most deaths related to the pandemic in Bangladesh. This has been mentioned in the methodology section of the manuscript. A sensitivity analysis has been performed to incorporate mortality data from all age groups, and it has been noted that there were no notable differences in the overall interpretation of the results.

6. In figure 1 please update the flow chart to show the exclusion of deaths that were outside of the study time frame.

**Response**: We have revised the flow-chart as suggested.
